# Causal discovery for the microbiome

**DOI:** 10.1016/S2666-5247(22)00186-0

**Published:** 2022-09-21

**Authors:** Jukka Corander, William P Hanage, Johan Pensar

**Affiliations:** Department of Biostatistics, Institute of Basic Medical Sciences, University of Oslo, Oslo, Norway; Parasites and Microbes, The Wellcome Sanger Institute, Cambridge, UK; Helsinki Institute for Information Technology, Department of Mathematics and Statistics, University of Helsinki, Helsinki, Finland; Center for Communicable Disease Dynamics, Harvard T.H Chan School of Public Health, Boston, MA, USA; Department of Mathematics, University of Oslo, Oslo, Norway

## Abstract

Measurement and manipulation of the microbiome is generally considered to have great potential for understanding the causes of complex diseases in humans, developing new therapies, and finding preventive measures. Many studies have found significant associations between the microbiome and various diseases; however, Koch’s classical postulates remind us about the importance of causative reasoning when considering the relationship between microbes and a disease manifestation. Although causal discovery in observational microbiome data faces many challenges, methodological advances in causal structure learning have improved the potential of data-driven prediction of causal effects in large-scale biological systems. In this Personal View, we show the capability of existing methods for inferring causal effects from metagenomic data, and we highlight ways in which the introduction of causal structures that are more flexible than existing structures offers new opportunities for causal reasoning. Our observations suggest that microbiome research can further benefit from tools developed in the past 5 years in causal discovery and learn from their applications elsewhere.

## Introduction

Microbiome science has found many associations linking the communities of microorganisms that live on and in humans with varying states of health and disease.^1^ However, further work to define the precise mechanisms by which microbiome communities influence health outcomes has been much more difficult.^2^ There are several reasons for this challenge, pre-eminently the problem of causation; we do not know whether the community produced the outcome, or whether the outcome selected for the community. Direction of causation is not the only obstacle that researchers have when seeking a biological mechanism from a collection of metagenomic datasets that show a difference between cases and controls, no matter how significant. Microbiome data are extremely high-dimensional, such that thousands of taxa might be in a sample, and the abundance of each taxon is often only assayed as proportions of the total, rather than absolute abundances. Whether only one of the taxa present is responsible, or the community as a whole, or a combination of both, is often unclear. These fundamental barriers are most readily overcome by experiments, such as the transfer of gut microbiomes to germ-free mice, and questioning whether this transfer leads to different outcomes, which reflects the basis of Koch’s classical postulates.^3^ Such studies are expensive, and although they can offer substantial insight on health outcomes, they might struggle to define the precise taxa or mechanisms involved because animal models do not generally enable accurate translation to human disease.^4^

The use of metagenomic non-experimental data from the microbiome to identify plausible causal interactions is desirable on multiple grounds. Firstly, it has the potential to identify plausible disease mechanisms and potential interventions. Secondly, even if causal discovery from non-experimental data is only partly possible, it can still reduce the number of experiments needed to securely assign the true causal factors. For example, if a causal discovery method highlights the individual taxa associated with the outcome, these can be tested separately, rather than the whole community. Finally, many causal discovery applications would require a framework able to handle the dynamic changes in composition that are thought to characterise many microbiome-mediated outcomes.^5^

We approach this problem by first defining terms for causal reasoning in the microbiome with the established framework of directed acyclic graphs (DAGs), and applying a method for causal discovery in high-dimensional cross-sectional data to accurately identify causal relationships in simulated metagenomic data. We then discuss an extension of standard causal graphs, labelled DAGs (LDAGs), which improve inference about the direction of causality.

## DAG for causal reasoning in the microbiome

**Figure 1:**
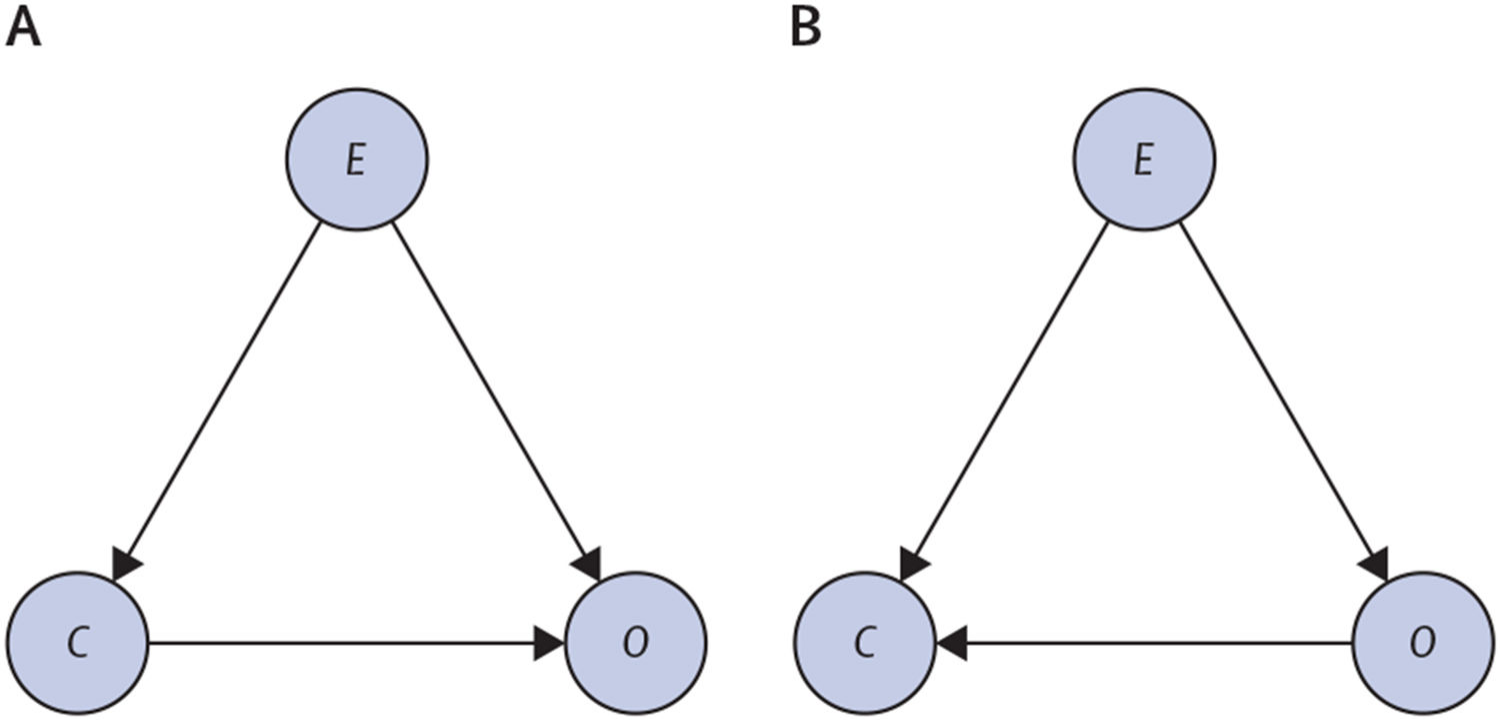
Two DAGs describing different causal structures The DAGs describe different causal structures (A, B) for a system involving a microbiome community (*C*), an outcome node of interest (*O*), and an environmental or confounding factor (*E*). The directed edges represent causal relationships between the variables. DAGs=directed acyclic graph.

The most widely established language for causal reasoning is that of DAGs.^6^ A causal DAG consists of system variables, shown as nodes, together with arrows between them illustrating causal relationships and their direction. Indirect and direct causes of the state of a variable can hence be readily communicated (figure 1). We started by defining three variables: the status of a microbiome community (*C*), an environmental factor *E*, and an outcome of interest *O*, which could be the presence of symptoms, or other measures (eg, BMI). Variable *C* might be defined at varying levels of resolution, from the presence of a specific species, or other operational taxonomic unit (OTU), to a specific community of OTUs. A major challenge for causal analysis of the microbiome and disease is the fact that the appropriate DAG must be learned from the data. Learning of DAGs is generally referred to as causal discovery from observational data. This step is a prerequisite for a statistical test of a causal hypothesis based on interventions, which can then be analysed with the *do* calculus pioneered by Pearl.^6^

Figure 1A shows a causal DAG structure, in which the edge *C* → *O* corresponds to the community, having a direct causal effect on the outcome. The environmental factor functions as a confounding factor, having a direct causal effect on the microbiome community and the disease status. By a causal effect, we mean any measure that can be calculated from *p*(*O*∣*do*[*C*]), where the *do* operator represents an intervention being done on C^.6^

With a DAG, such as one of the graphs shown in figure 1A and a set of observational data, consistently estimating the causal effects between the variables is possible (figure 1A).^6^ We can rewrite the distribution of *O* under an intervention on *C* as:

This formula adjusts for the confounding factor *E*,

$$p(O|\text{do}[C])=\sum_{E}p(O|C,E)p(E)$$

which corresponds to blocking all backdoor paths (ie, the non-causal path *C* ← *E* → *O*; figure 1) in the graph. Of note, the probabilities on the right-hand side in the expression do not contain the *do* operator; therefore, they do not imply an intervention and can be estimated from observational data. Similar treatment of the DAG shown in figure 1B, in which there is no effect of the microbiome community on the presence of the outcome, yet the two remain correlated because of causation in the reverse direction, yields: *p*(*O*∣*do*[*C*])=p(*O*).

Although DAGs can be inferred from observational data with conditional independence statements, this is only possible up to the Markov equivalence class, which is the set of DAGs that contains the same dependence structure. For example, the DAGs in figure 1 are in the same Markov equivalence class but have very different causal implications, which is a formal way of stating the familiar problem in distinguishing cause from correlation. We can illustrate the problem using a method, known as intervention calculus when the DAG is absent (IDA), specifically developed for the analysis of high-dimensional data, such as networks of gene expression, in which the DAG is initially unknown.^7,8^ IDA both learns the Markov equivalence class and then estimates the possible causal effects for each DAG within it. IDA needs to make rather strong assumptions that the joint distribution over the variables in the considered system is multivariate normal (with a DAG-based covariance structure) and that there are no hidden confounding variables. Even if these assumptions are unlikely to be completely fulfilled by real datasets, proof-of-concept results have already shown the usefulness of IDA (and a variant of it) on large-scale biological systems involving gene expression data.^8,9^ We further illustrate the potential of IDA and its limitations for causal inference from microbiome data in a controlled setting, with simulations reflecting an ideal situation in which the assumptions are valid.

## Causal inference from cross-sectional microbiome data

**Figure 2:**
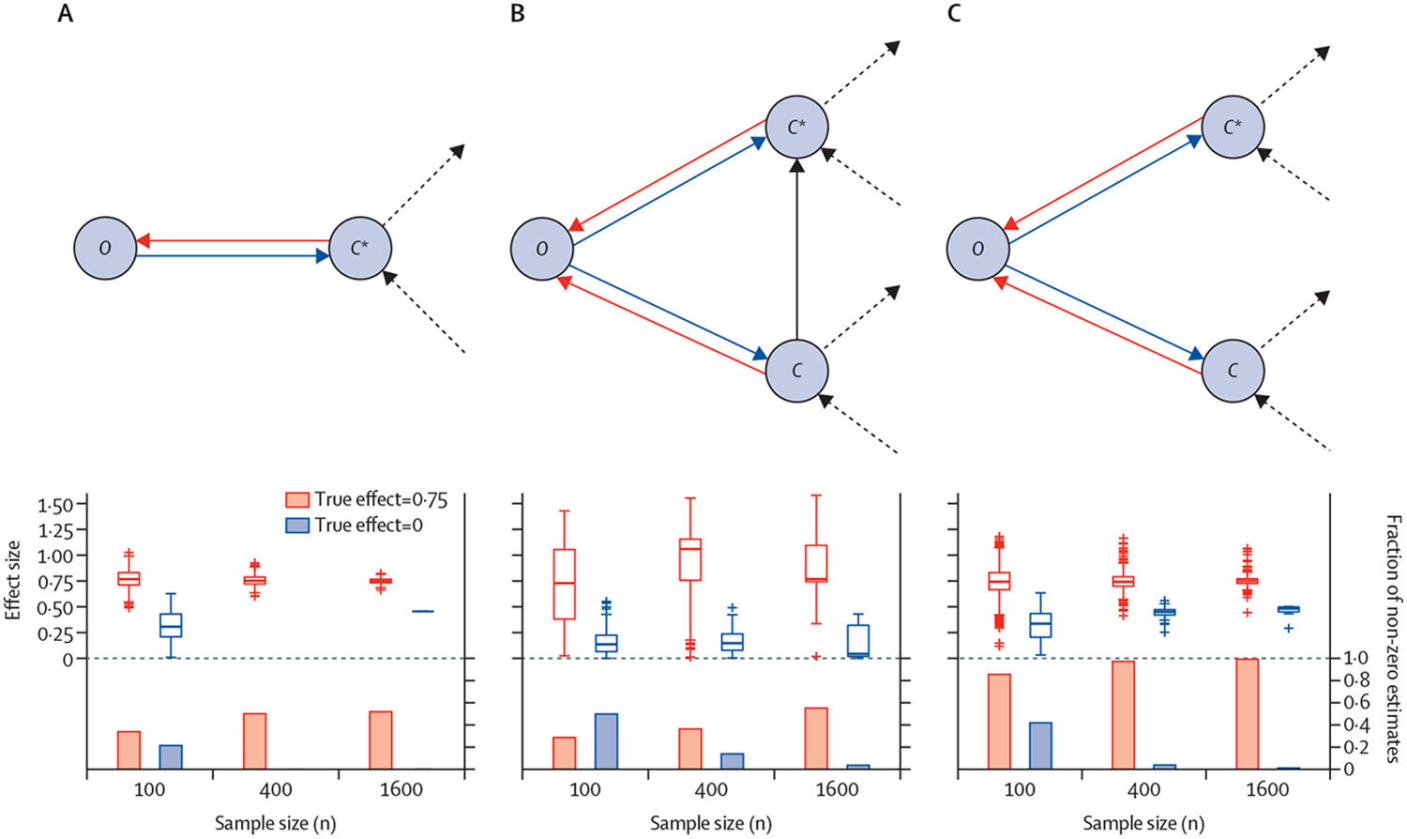
Lower bound of the causal effect of on Causal effect estimated by intervention calculus when the directed acyclic graph is absent under three different scenarios (A–C) illustrated by the graph structures. The red arrows represent the causal mechanism in which the microbiome affects the outcome status (true effect=0·75) and the blue arrows represent the reversed causal mechanism in which the outcome status affects the microbiome (true effect=0). The dashed arrows represent potential interactions between *C*, C*, and the rest of the operational taxonomic units. The box–bar plots summarise the results of the simulations obtained for different sample sizes shown on the horizontal axis. The bars show the proportion of non-zero estimates (right vertical axis) and the boxes show the distributions of the of the non-zero estimates. *C*=microbiome community.

We considered a single outcome node *O* and 100 OTUs, *C*_1_ to *C*_100_, which has a coexistence pattern described by a DAG-based multivariate normal distribution. The OTU nodes represent relative abundances, mimicking log-transformed and normalised titre values on the basis of either 16S rRNA or shotgun metagenomics, and the outcome node represents a continuous valued phenotype of interest. The simulated system was specified to model symbiotic relationships of varying strength between pairs of OTUs, corresponding to positive correlations between OTU abundances, but we could equally have allowed for antagonistic relationships, or negative correlations. The aim of the experiment was to estimate the causal effect of a randomly chosen OTU C* (ie, *C*_1_ to *C*_100_) on the outcome node. The sample size in any of these experiments refers to the number of people from which metagenomic profiles are available. The structure of the DAG for the OTUs was generated randomly, such that the number of neighbours of an OTU was three, and we considered three different scenarios (figure 2) for the link between the outcome node and the microbiome. In the simplest scenario in figure 2A, the outcome node is directly linked to a single OTU. The other two scenarios in figure 2B, C extend this causal dependence to a simple community consisting of two OTUs that are either directly linked (figure 2B) or not directly linked (figure 2C). For each scenario, we considered both the situation in which the microbiome is a cause of the outcome node and the reversed situation in which the outcome is affecting the status of the microbiome, which allowed us to assess the accuracy of IDA both in terms of true positives and false positives. For each scenario, the presented results are averages from 1000 randomly generated models. Since IDA outputs a set of estimates, we summarised the output by the minimum absolute value, which can be considered a lower bound on the causal effect. We used the R package (version 2.7) pcalg to generate the random DAGs and run the IDA algorithm.^10^

The results of the experiments are summarised by the box–bar plot in figure 2. In the first scenario (figure 2A), we see that IDA reaches a true discovery rate of around 50% for a sample size of 400; however, the rate does not improve when increasing the sample size to 1600. In fact, the rate would not improve substantially even with access to infinitely large samples. The theoretical upper limit on the discovery rate, which was 52·6% in this simulation, depends on the specific structures in the randomly generated systems; to infer the direction of C* → *O*, the algorithm exploits the direction of other edges. More specifically, an incoming edge to C* from any other OTU must exist, for the statistical reasons that we discuss later in this Personal View. From the biological perspective, the outcome and OTU covariation have an indistinguishable pattern under either direction of causality in the first scenario (figure 2A). Other notable observations are that the magnitude of the causal effect is quite accurately estimated for the non-zero effects, and that the false positive rate is low for all sample sizes greater than 100.

In the second scenario (figure 2B), the theoretically optimal true discovery rate was 83·6%; however, IDA clearly requires larger samples to infer the slightly more complex link between the outcome node and the microbiome, which is reflected not only by a lower relative true discovery rate, but also by an increase in the false discovery rate. Additionally, there is an increased uncertainty in the estimation of the magnitude of the causal effect. For consistent estimation of the causal effect, the method must not only infer the direction of C* → *O*, but also *C* → C*, since the backdoor path C* ← *C* → *O* otherwise remains open, introducing bias to the estimate.

The third scenario (figure 2C) represents a situation in which consistent causal discovery of the C* → *O* mechanism is possible regardless of the structure of the rest of the system because C* → *O* ← *C* forms a so-called v-structure, in which C* and *C* are not directly connected. In biological terms, this v-structure corresponds to two causal OTUs which have relative abundances that are not directly influencing each other. In contrast to the previous scenarios, IDA reaches a true discovery rate close to 100%, with a very low false discovery rate, already for a sample size of 400. Compared with the first scenario (figure 2A), the uncertainty in estimating the magnitude of the effect is slightly higher, which is the result of backdoor paths from C* to *O* via *C* that are incorrectly inferred to be open, introducing bias to the estimate.

These simulations (figure 2) highlight both the potential and limitations of DAG-based causal discovery. An obvious limitation is the rather strong assumptions, which are difficult to verify in practice, yet necessary for the approach to work, even in theory. However, the simulations show that when we have a situation in which the assumptions are fulfilled, IDA can be quite accurate for samples of a size that could be experimentally achieved, at least when compared with the theoretically optimal limit. This point brings us to another critical limitation: whether or not causal discovery is possible ultimately depends on the structure of the actual system. Again, this limitation is not strictly because of a problem in the DAG-based approach, but rather a more fundamental issue highlighting the insufficiency of observational data when it comes to drawing causal conclusions. Nonetheless, IDA and similar types of methods can provide a useful tool for doing an initial causal analysis on observational microbiome data, leading to hypotheses that can be attempted to be verified experimentally.

## LDAGs to improve causal reasoning

The class of LDAGs introduces a general representation of context-specific independence in Bayesian networks.^11^ An LDAG adheres to the Markov properties of traditional DAGs; however, in addition to conditional independence, it encodes particular context-specific independence statements through labels attached to the edges. If the context of a label is satisfied, the dependence conveyed by the associated edge is removed. In other words, LDAGs provide a more expressive class of dependence structures than traditional DAGs.

The application of LDAGs is illustrated by an analysis of *Clostridioides difficile* associated diarrhoea (CDAD), which is a microbiome mediated disease for which we have relatively secure causal understanding. Although *C difficile* is often found in the microbiome of healthy people, it is present in small quantities. However, after disruption of the gut microbiome community by antibiotic treatment, *C difficile* can grow to a higher titre, resulting in diarrhoea.^12^ Although the infection can be treated with antibiotics, *C difficile* forms hard, resistant spores that can survive the treatment, and so the disease becomes chronic and resumes when antibiotics are withdrawn. To emphasise this situation as a case of a microbiome-mediated disease in which the community is important, CDAD can be successfully treated with faecal microbiome transplants from healthy individuals.^13^

**Figure 3:**
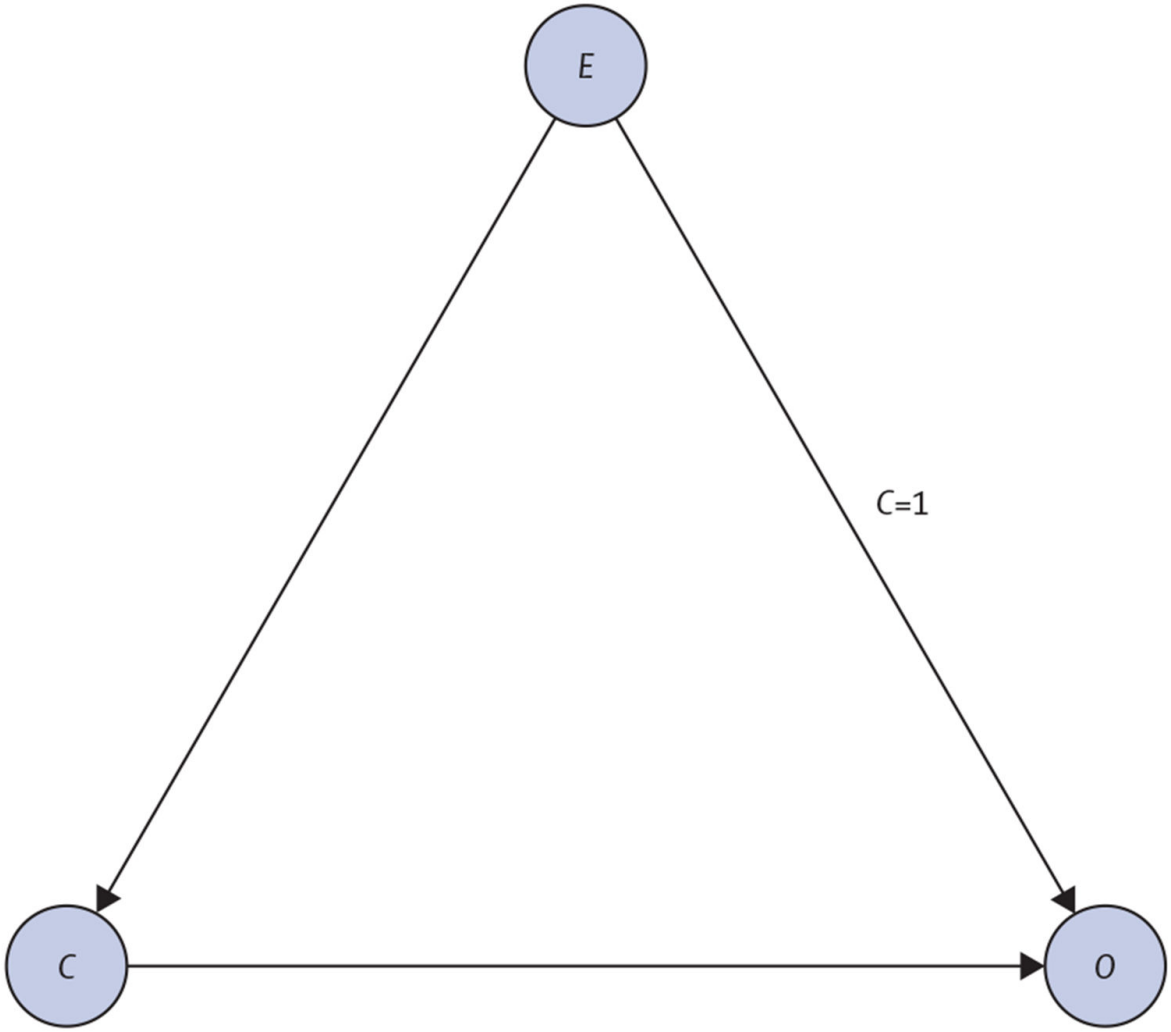
A labelled directed acyclic graph describing the causal structure of *Clostridioides difficile* *C* represents a person’s microbiome community, which is either dominated (*C*=1) or not dominated (*C*=0) by *C difficile; E* represents whether or not the person receives antibiotic treatment. *O* represents whether or not the person has persistent diarrhoea. The directed edges represent causal relationships between the variables; and the label implies that the causal direct effect (*E* to *O*) vanishes when the microbiome community is dominated by *C difficile* (ie, when *C*=1).

The causal structure of CDAD can be described by an LDAG in figure 3. Although a DAG can represent the cause of diarrhoea, it hides the explicit role of the antibiotic treatment (*E*) in irreversibly altering the microbiome community (*C*), which is encoded by the context-specific independence represented as the label on the edge from antibiotic treatment to the persistent diarrhoea in the LDAG. Assuming that we are given observational data from this system and that we have previous knowledge of *E* being a direct cause of *C* and *O* (ie, *C* ← *E* → *O*), from the data we can infer the presence of an edge between *C* and *O*, but not the direction of the edge, which translates into an ambiguity about whether the observed microbiome status causes the disease or is a consequence of the disease. From a structure learning perspective, we cannot establish the direction of the final edge between *C* and *O*, since the two possible DAGs (figure 1A, B) belong to the same Markov equivalence class. However, with LDAGs, the existing context-specific independence would provide enough information to deduce the direction of the final edge. For *C* to be able to affect the relationship between *E* and *O*, it must be a cause of either *E* or *O*, and since we know that *C* is an effect of *E*, it must consequently be a cause of *O* (ie, *C* → *O*).

**Figure 4:**
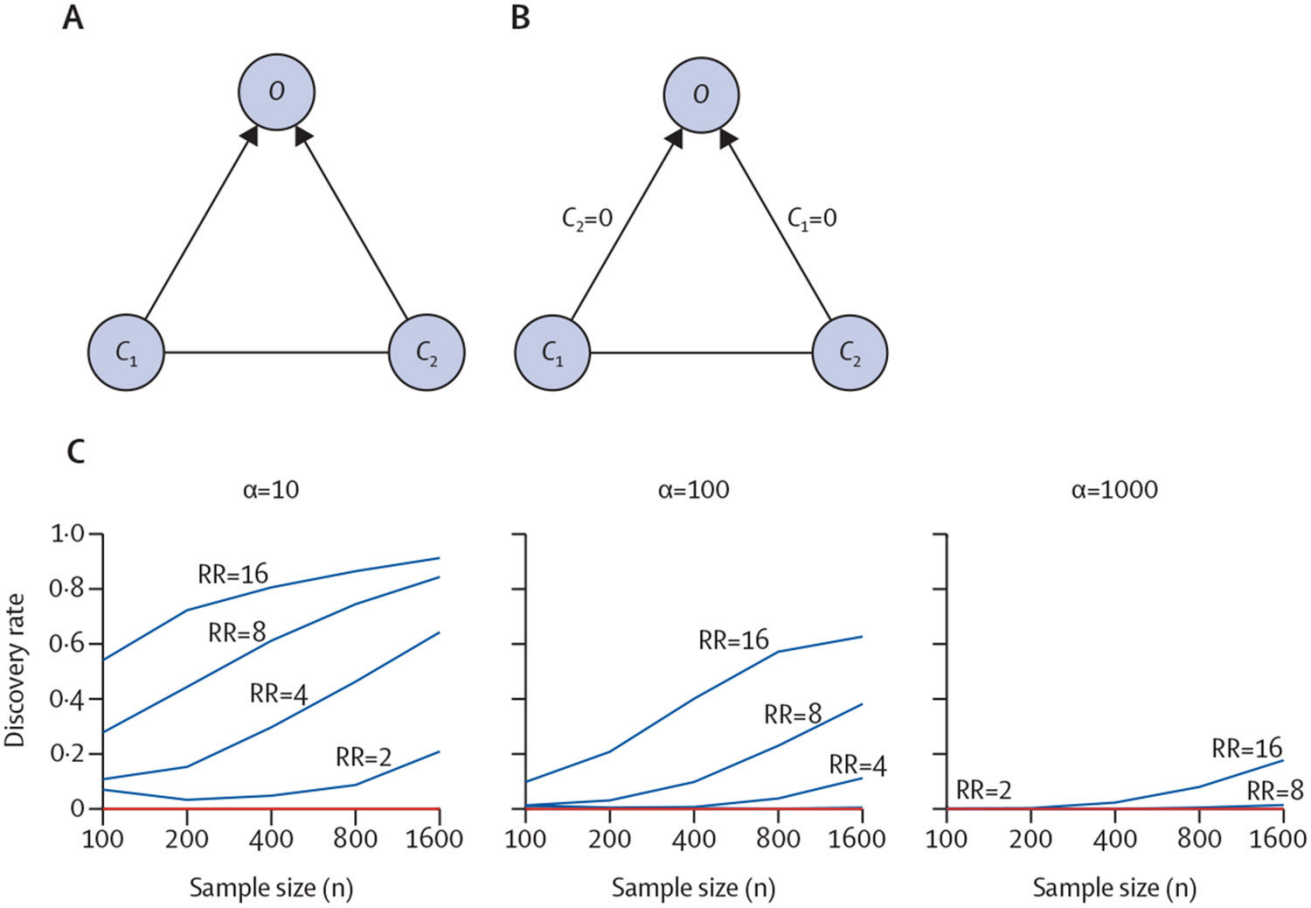
Causal structures and results of the LDAG simulation The discovery rate (vertical axis) is the proportion of cases in which either method successfully discovered the causal mechanism: LDAG-based method (blue), DAG-based method (red). The different plots correspond to different a-values and the different curves within each plot correspond to different RRs. *C*=microbiome community. DAG=directed acyclic graph. LDAG=labelled DAG. *O*=outcome node of interest. RR=risk ratio.

LDAGs are also capable of detecting causal interactions that arise when multiple taxa are necessary for an outcome. Although much recent work on the microbiome has involved narrowing down mechanisms to individual taxa, the ability to establish the effects of communities, in particular emergent properties, would be a great advantage.^2^ We modelled an intentionally simple example of community effect using just two OTUs, *C*_1_ and *C*_2_ (figure 4). Both OTUs are direct causes of a disease *O* and are marginally dependent, either directly by an edge or indirectly by an unobserved confounding factor, which is shown by the undirected line between them (figure 4A). In this simple example, we defined the OTUs as either not present (0) or present (1), and defined the outcome *O* as either asymptomatic (0) or symptomatic (1). Our aim was to infer the causal mechanism *C*_1_→ *O* ←*C*_2_ from observational data, without any prior information. As explained earlier, conventional DAGs cannot be used in this scenario. However, if the joint causal effect of *C*_1_ and *C*_2_ is only seen when both OTUs are present, an LDAG can correctly represent and estimate it from the data (figure 4B).

Using simulations, we generated the joint distribution of *C*_1_ and *C*_2_ so that the OTUs showed a coexistence pattern favouring configurations in which both or neither of the OTUs were present. We modelled the tendency of *C*_1_ and *C*_2_ to cooccur with a tuning parameter α; as α increases, the two OTUs are more likely to be found together (for example, because of cross feeding or other metabolic interactions).^13^ We set the baseline probability of developing the disease to 0·05 and examined the combined effect of relative risk or risk ratio and the coexistence tendency α, on the true causal discovery rate for various sample sizes under both the DAG-based and LDAG-based framework. The causal structures of the respective model class were evaluated with the Bayesian score,^11^ after which the highest scoring equivalence class was selected. If all structures in the inferred equivalence class contained the causal mechanism *C*_1_ → *O* ← *C*_2_, the result was considered correct, otherwise it was considered incorrect. For each setting, we generated 1000 models and corresponding datasets for which the results were summarised as discovery rates.

As expected, the true discovery rate of the DAG-based approach is zero (figure 4C), whereas the LDAG-based method successfully learns the underlying causal structure with an increasing probability as a function of the sample size and risk ratio. Nevertheless, the discovery rate decreases dramatically when the α-parameter is also increased, which is a consequence of its effect on the distribution over *C*_1_ and *C*_2_. In particular, as the parameter tends to infinity, the dependence between *C*_1_ and *C*_2_ becomes perfect (or deterministic), which corresponds to a situation with only one binary cause *C*=(*C*_1_, *C*_2_), and we cannot infer whether *C* → *O* or *C* ← *O*, since the two DAGs are Markov equivalent.

## Discussion

In this Personal View, we have discussed causal discovery using the DAG-based framework, in which one attempts to characterise the Markov equivalence class of causal structures from observational data. We have shown that identifiability of a causal effect of interest generally requires the direction of additional edges (figure 2A, B), but might not be possible in many cases, since characterising the Markov equivalence from observational data requires specific information of the direction of additional edges involving the relevant community or the diseases. However, we also showed that DAGs can have potential for microbiome data, as shown by the scenario in which two OTUs, which have relative abundances that are not directly connected, both have a causal effect (figure 2C). Consistent causal discovery is possible no matter the direction of the rest of the dependencies, provided that the biases in the measurement process or the assumed quantitative model do not obscure the true effects.

We showed that by switching to a more expressive class of models than DAGs, such as LDAGs, we can extract more information from the data, which can subsequently improve inference about the direction of causality. The advantage of LDAGs is the ability to encode context-specific independencies, such as the effect of antibiotic use in CDAD. We also showed that LDAGs can detect causal interactions that arise when multiple taxa are necessary for an outcome. In this Personal View, we focused on the scenario in which two taxa are required for disease, relevant to a circumstance in which the pathology is caused by multiple factors in the community. This causal network is readily and reliably identified depending on the sample size and the risk ratio, as we might expect from intuition. However, more crucially, if the causal taxa tend to coexist in the same community, our ability to detect the true effect and direction of causation is effectively reduced (figure 4C).

A critical assumption made throughout this work is that there are no hidden confounding factors, which is one of the reasons warranting the experimental approach to causal effect estimation, since they can be accounted for by appropriate randomisation procedures. We are also only considering statistical issues of the estimator (bias and variance) in a setting in which the assumptions are satisfied, and not potential problems (eg, contamination) with sample preparation and contamination, which are very important,^14,15^ but beyond the scope of this work. Generally, predictions based on purely observational data should not be seen as a replacement of intervention experiments, but they should instead be used as a tool for generating causal hypotheses that can guide in the design of follow-up experiments.

Other methods exist besides the ones we have considered and deserve attention. In particular, Mendelian randomisation is a popular and widely used instrumental variable method that exploits genetic variation as an instrument for analysing the causal effect of an exposure on an outcome of interest.^16,17^ Under some key instrumental variable assumptions, including the existence of a genetic variant associated with the exposure (ie, the instrument), the method is able to reduce both reverse causation and confounding, yet violations of the assumptions might lead to severe biases.^18^ The linear non-Gaussian acyclic model algorithm offers another interesting structure learning alternative that guarantees identifiability of the whole DAG under the specified assumptions.^19^ Other alternative causal inference methods include the cavity method for dynamic physical systems,^20^ and approximate Bayesian computation-based inference for statistical simulator models.^21^ All of these methods can complement the approach we have described.

The focus of this Personal View has been on cross-sectional data, which is more abundant than longitudinal data since longitudinal studies are more expensive and time consuming than cross-sectional studies. However, the DAG-based framework can also be extended to the longitudinal setting by introducing time-specific variables;^22,23^ in fact, the temporal ordering of the variables might even facilitate detection of causal relationships from longitudinal data.^24^ Finally, a particularly beneficial class of methods are those that combine observational and experimental data. In the structure learning framework, intervention experiments improve identifiability by narrowing down the class of possible causal structures.^25-27^ A method exploiting a general invariance principle of causal systems was developed and applied to gene expression data,^28,29^ which contained both observational samples and interventional samples obtained through perturbation by single gene knockouts. Provided that a plausible animal model of the disease is available, and one can systematically knock out components of the microbiome community, this kind of approach might open up new frontiers.

For simplicity, we focused on the original IDA method in the high-dimensional OTU simulation. However, since the pioneering work by Maathuis and colleagues,^7,8^ a line of research has given rise to several extensions and improvements of the original method, and we expect to see further methodological developments in the near future. Previous advancements include extending IDA to joint interventions,^30^ improving its accuracy by more careful selection of adjustment sets,^31,32^ and improving its scalability through local structure learning.^33^ In addition, one of the main limitations of the original IDA method is its restricted ability to account for the typically considerable amount of uncertainty involved in inference procedure. To address this restriction, IDA has been combined with frequentist resampling techniques,^9,34^ and also extended to the Bayesian setting.^35,36^ The Bayesian approach, in particular, has shown promising results in terms of accuracy, albeit is currently limited to medium-sized systems for computational reasons. There have also been several advancements in identification of valid adjustment sets in causally insufficient systems, thus enabling causal effect estimation in some cases when latent confounders are allowed.^37,38^ Although such a setting is more realistic for most real-world scenarios, it is also considerably more challenging because of issues related to identifiability and computational complexity, and practical applications are, therefore, still scarce, especially in the high-dimensional setting.

Our consideration of the causal discovery problem focused on the core challenge of identifying causal relationships between OTUs and outcomes of interest with metagenomics. However, increase in the use of multiple omics in microbiome research, such as metatranscriptomics and metabolomics in conjugation with metagenomics,^39^ suggest that more precise causal discovery could be made by developing hierarchical models that are capable of integrating such data and feeding relevant, existing biological knowledge about possible directions of system variable relationships into the inference process. We have implicitly emphasised situations with high biomass samples, as is typical with gut microbiome studies. Low biomass settings, such as lung and skin, will come with their own specific challenges because generating robust molecular data from such samples is generally more difficult. However, the level of microbial complexity tends to be lower in low biomass settings than in the gut, which would generally reduce the risk of discovering spurious causal relationships because the curse of dimensionality would be substantially reduced compared with the gut microbiome.

Overall, we conclude that combining better means of characterising causal structures, together with experimental models of disease, will help to make the future of the microbiome research even brighter than the present.
